# Recurrence of Antineutrophil Cytoplasmic Antibody (ANCA)-Associated Glomerulonephritis Following Kidney Transplantation: A Case Report With an Institutional Registry Review From Saudi Arabia

**DOI:** 10.7759/cureus.111490

**Published:** 2026-06-25

**Authors:** Sumayah Askandarani, Khadija M Alshehabi, Khalid Akkari, Abdulaziz S Alghamdi, Baher M Algadaa, Najla Aldaoud, Abdulnaser Alabadi

**Affiliations:** 1 Multi-Organ Transplant Center, King Fahad Specialist Hospital, Eastern Health Cluster, Dammam, SAU; 2 Department of Nephrology, Salmaniya Medical Complex, Government Hospitals, Manama, BHR; 3 Department of Pathology and Laboratory Medicine, King Fahad Specialist Hospital, Eastern Health Cluster, Dammam, SAU

**Keywords:** anca-associated vasculitis, crescentic glomerulonephritis, graft dysfunction, immunosuppression, kidney transplantation, recurrence

## Abstract

Background

Recurrence of antineutrophil cytoplasmic antibody (ANCA)-associated vasculitis (AAV) following kidney transplantation is uncommon but may result in significant graft dysfunction and poor clinical outcomes. Data regarding recurrence after transplantation remains limited, particularly in the Middle East.

Methods

We present a case of early recurrent ANCA-associated glomerulonephritis following kidney transplantation, supported by a retrospective review of our institutional kidney transplant registry at King Fahad Specialist Hospital in Dammam, Saudi Arabia. Between 2008 and 2026, six patients underwent kidney transplantation for AAV-related end-stage kidney disease among 2306 kidney transplant recipients.

Results

Among 2306 kidney transplantations performed during the study period, six patients underwent transplantation for AAV-related end-stage kidney disease. Recurrence occurred in one patient (16.7%) and presented early in the post-transplant period with severe graft dysfunction. Histopathological evaluation demonstrated crescentic glomerulonephritis consistent with recurrent disease. Despite treatment with pulse corticosteroids and plasma exchange, the patient's clinical course was complicated by severe infectious complications resulting in death. The remaining five patients demonstrated generally favorable graft outcomes without evidence of recurrence during follow-up.

Conclusion

Recurrence of AAV after kidney transplantation remains uncommon. Although only one recurrence was identified in our cohort, the disease followed an aggressive clinical course in this patient, underscoring the importance of early recognition and prompt histological confirmation. Larger multicenter studies are needed to better define recurrence risk and optimize management strategies.

## Introduction

Antineutrophil cytoplasmic antibody (ANCA)-associated vasculitis (AAV) comprises a group of systemic small-vessel vasculitides, including granulomatosis with polyangiitis (GPA) and microscopic polyangiitis (MPA) [1,2]. Renal involvement is common and typically manifests as pauci-immune crescentic glomerulonephritis, representing a major determinant of morbidity and mortality [2,3]. Despite advances in immunosuppressive therapy, a substantial proportion of patients ultimately progress to end-stage kidney disease (ESKD), particularly those presenting with severe renal involvement at diagnosis [1-4].

Kidney transplantation is the preferred renal replacement therapy for patients with ESKD secondary to AAV, offering improved survival and quality of life compared with dialysis [5-7]. The Dutch Transplantation in Vasculitis (DUTRAVAS) study reported one-year and five-year graft survival rates of 94.5% and 82.8%, respectively, which were comparable to those reported in the general kidney transplant population in Europe and North America [5], supporting kidney transplantation as an effective treatment option for patients with AAV undergoing transplantation during disease remission. Current recommendations advise proceeding with transplantation after achieving sustained clinical remission, typically for at least six months, regardless of ANCA serostatus [6,8].

Despite these favorable outcomes, recurrence of AAV following transplantation remains an important clinical concern. In the modern era, recurrence is relatively uncommon, with reported rates ranging from approximately 3% to 10% [6,9,10]. Recurrence may occur at any time after transplantation, ranging from early presentations within weeks to late relapses occurring years later [10-12]. Early recurrence is often aggressive and may present with delayed graft function, whereas late recurrence tends to follow a more indolent course [10-12].

Diagnosis is challenging due to overlap with other causes of graft dysfunction, including but not limited to rejection and infection. Definitive diagnosis relies on allograft biopsy demonstrating pauci-immune necrotizing and crescentic glomerulonephritis [11,12].

Given the rarity and heterogeneity of post-transplant AAV recurrence, most available evidence is derived from retrospective studies and case reports; therefore, optimal strategies for risk stratification, prevention, and management remain incompletely defined [6,8-10]. In addition to the existing literature, single-center experiences may provide valuable real-world insights.

We report the post-transplantation outcomes of six patients with biopsy-proven AAV who underwent kidney transplantation between 2008 and 2026 at King Fahad Specialist Hospital, a high-volume transplant center in Dammam, Saudi Arabia. In addition to our center's experience, we provide a comprehensive literature review focusing on the diagnostic challenges, management strategies, and clinical outcomes associated with AAV in the transplant setting.

## Materials and methods

Study design and setting

This manuscript comprises a detailed case report of recurrent ANCA-associated glomerulonephritis following kidney transplantation, supported by a retrospective review of the institutional kidney transplant registry at King Fahad Specialist Hospital in Dammam, Saudi Arabia. The registry review was performed to identify all patients who underwent kidney transplantation for ESKD secondary to AAV between January 2008 and January 2026, thereby providing institutional context regarding the frequency of disease recurrence and transplant outcomes.

Inclusion and exclusion criteria

Patients were eligible if they were aged ≥18 years and diagnosed with AAV as the primary cause of ESKD prior to kidney transplantation. Diagnosis of AAV was established based on compatible clinical presentation, ANCA serology, and/or kidney biopsy findings.

Patients were excluded if they had incomplete clinical or follow-up records and an uncertain primary kidney disease diagnosis or underwent multi-organ transplantation.

Data collection

Clinical, laboratory, pathological, and transplant-related data were obtained from electronic medical records, transplant databases, pathology archives, and laboratory information systems. Collected variables included demographic characteristics, AAV subtype, ANCA serology, remission status at transplantation, donor characteristics, immunosuppressive regimens, recurrence characteristics, biopsy findings, graft function, and patient outcomes.

Definitions and outcomes

Recurrence was defined as biopsy-proven pauci-immune necrotizing and/or crescentic glomerulonephritis involving the renal allograft in the appropriate clinical context.

The primary outcome was recurrence of AAV following kidney transplantation. Secondary outcomes included graft function, graft survival, patient survival, and post-transplant complications.

Statistical analysis

Due to the small sample size, descriptive statistics were used to summarize baseline characteristics, recurrence rates, graft outcomes, and patient outcomes.

## Results

A total of six patients with AAV underwent kidney transplantation during the study period. Among these, one patient (16.7%) developed recurrence of AAV following transplantation. The recurrence occurred early in the post-transplant period and was associated with a severe clinical course.

The remaining five patients did not demonstrate evidence of disease recurrence during follow-up. Most maintained stable graft function, while one patient developed chronic allograft nephropathy. Overall outcomes were favorable in non-recurrent cases. Baseline demographic, clinical, and transplant-related characteristics are summarized in Table 1.

**Table 1:** Clinical characteristics and outcomes of patients with AAV undergoing kidney transplantation at King Fahad Specialist Hospital, Dammam (2008-2026) C-ANCA: cytoplasmic antineutrophil cytoplasmic antibodies; P-ANCA: perinuclear antineutrophil cytoplasmic antibodies; AZA: azathioprine; PRA: panel reactive antibodies; MM: mismatch; DSA: donor-specific antibodies; IS: immunosuppressive agents; AMR: antibody-mediated rejection; PJP: *Pneumocystis jirovecii* pneumonia; DVT: deep vein thrombosis; GN: glomerulonephritis; ESKD: end-stage kidney disease; HLA: human leukocyte antigen; AAV: ANCA-associated vasculitis

Age at transplant (year)	ANCA type	Organ involvement	Therapy	Duration of vasculitis before ESKD	Period of remission before transplant (years)	ANCA titer at transplant	Donor type	Pre-formed DSA	HLA mismatch	Induction	IS	Complications post-transplant (first year)	Kidney biopsy	Recurrence	Outcome
21	P-ANCA	Kidney, heart, chest	Rituximab, plasma exchange	1 month	2	Negative	Living related	None	4/8 MM, no DSA, negative cross-match	Thymoglobulin	Triple	AMR with de novo DSA, perinephric infected hematoma + PJP	AMR	No	Chronic allograft nephropathy
58	P-ANCA	Kidney	Cyclophosphamide, steroid, maintained on AZA	1 year	2	Negative	Deceased donor	None	6/8 MM, no DSA, negative cross-match	Thymoglobulin	Triple	None	None	No	Good
31	ANCA	Kidney	Cyclophosphamide, plasma exchange	1 month	1	Negative	Living related	None	3/8, no DSA, negative cross-match	Thymoglobulin	Triple	None	None	No	Good
64	C- ANCA	Kidney	Cyclophosphamide, steroids, rituximab	2 years	2	Negative	Living unrelated (paired exchange)	None	3/8 MM, no DSA, negative cross-match	Thymoglobulin	Triple	Early ANCA recurrence, retroperitoneal hemorrhage, septic shock	Crescentic GN	Yes	Early recurrence and death
17	C- ANCA	Kidney	Undocumented	Undocumented	7	Negative	Living related	None	2/8 MM, no DSA, negative cross-match	Basiliximab	Triple	None	None	No	Good
63	ANCA	Kidney	Undocumented	1 month	2	Negative	Living unrelated (paired exchange)	High PRA	7/8 MM, no DSA, negative cross-match	Thymoglobulin	Triple	Intraoperative bleeding, complicated by extensive lower limb DVT	None	No	Good

Case presentation

A 64-year-old female patient with a history of longstanding hypertension developed ESKD secondary to AAV, initially diagnosed in 2021 as pauci-immune crescentic glomerulonephritis. She was initially treated with corticosteroids and rituximab without adequate response, followed by cyclophosphamide, which achieved clinical remission with the normalization of ANCA titers. Due to progressive renal dysfunction, she commenced hemodialysis via an arteriovenous fistula in 2023.

The patient underwent a living-unrelated kidney transplantation through a paired exchange program on August 18, 2025. Pre-transplant evaluation confirmed clinical remission. Immunological workup demonstrated 3/8 human leukocyte antigen (HLA) mismatch, negative donor-specific antibodies, and negative T- and B-cell flow cytometry cross-matches. Induction immunosuppression consisted of thymoglobulin (3.9 mg/kg), followed by maintenance therapy with prednisolone, tacrolimus, and mycophenolate mofetil, in addition to standard antimicrobial prophylaxis.

The early postoperative course was complicated by slow graft function, with serum creatinine plateauing at 280 μmol/L. Urinalysis revealed hematuria (+++), proteinuria, and a urine albumin-to-creatinine ratio of 332 mg/g. Doppler ultrasound demonstrated a well-perfused graft with normal resistive indices and no evidence of obstruction or vascular compromise.

**Figure 1:**
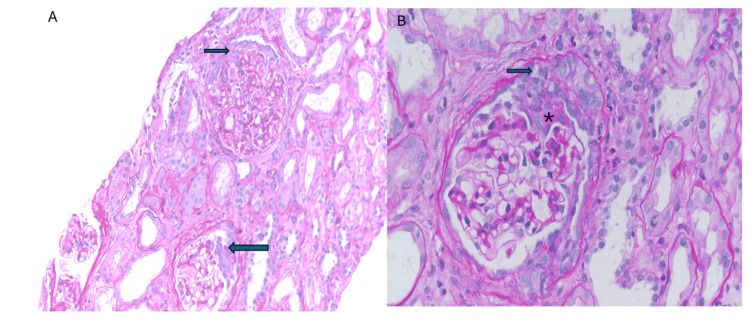
Histopathological findings of recurrent ANCA-associated glomerulonephritis (A) Medium-power magnification showing two glomeruli with early crescent formation (arrows) (PAS, ×100). (B) Medium-power magnification showing crescent formation (arrow) with fibrin deposition (star) (PAS, ×200). ANCA: antineutrophil cytoplasmic antibody; PAS: periodic acid-Schiff

**Figure 2:**
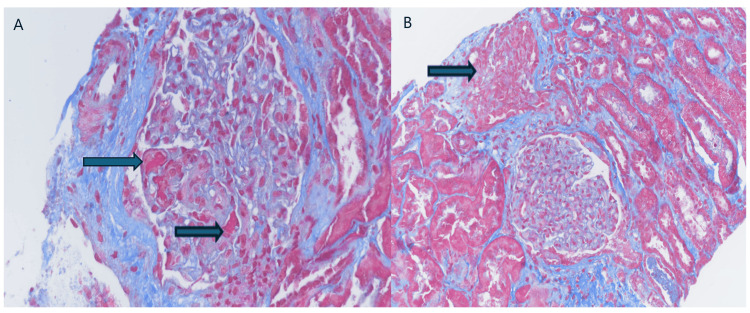
Histopathological findings of recurrent ANCA-associated glomerulonephritis (A) High-power magnification showing fibrin deposition (arrows) (Masson trichrome, ×400). (B) High-power magnification showing cellular crescent formation filling Bowman's space (arrow) (Masson trichrome, ×400). ANCA: antineutrophil cytoplasmic antibody

Given persistent graft dysfunction, an allograft biopsy was performed on postoperative day 9. Histopathological examination revealed early crescent formation in several glomeruli without significant endocapillary proliferation, raising concern for recurrence of AAV as shown in Figure 1A-1B and Figure 2A-2B. Serological testing demonstrated positive c-ANCA, while anti-glomerular basement membrane antibodies were negative.

The patient was treated with intravenous methylprednisolone (500 mg daily for three days) followed by plasma exchange (seven sessions). An initial improvement in renal function was observed. Rituximab was planned as part of further management; however, this was deferred due to subsequent clinical deterioration.

The clinical course was complicated by bacteremia with extended-spectrum beta-lactamase (ESBL)-producing *Klebsiella pneumoniae* secondary to cellulitis, necessitating intravenous antibiotics and reduction of immunosuppression. The patient subsequently developed internal jugular vein thrombosis requiring anticoagulation. Her condition further deteriorated with septic shock due to hospital-acquired pneumonia and hemorrhagic shock from retroperitoneal bleeding, resulting in multi-organ failure. Despite intensive care support, including vasopressors and mechanical ventilation, the patient died.

## Discussion

AAV remains an important cause of ESKD, with approximately 20-40% of affected patients ultimately requiring kidney replacement therapy despite advances in immunosuppressive treatment [2,3]. Kidney transplantation is considered the preferred treatment for patients who reach ESKD, providing excellent long-term graft and patient survival when performed during sustained disease remission [5-7]. Nevertheless, recurrence of AAV after transplantation, although uncommon, remains a clinically important complication because it may jeopardize graft function and patient survival.

Our institutional registry review identified six patients who underwent kidney transplantation for AAV over an 18-year period, of whom one (16.7%) developed biopsy-proven recurrent disease. Although this recurrence rate appears higher than those reported in larger cohorts (3-10%) [6,9,10], this finding should be interpreted cautiously given the very small sample size. Importantly, our patient developed an early and aggressive recurrence associated with severe infectious complications and death, whereas the remaining five patients maintained favorable graft outcomes without recurrence, consistent with previous reports demonstrating good transplant outcomes in patients transplanted during disease remission [5,6].

Although the present study focuses on a Saudi transplant population, regional epidemiological data on AAV remain limited. Few studies from the Gulf region have characterized the clinical phenotype and serological profile of AAV, and no comparable kidney transplant cohorts have been reported from Saudi Arabia. A recent multicenter study from the United Arab Emirates (UAE) reviewed the available Gulf literature and reported that Saudi cohorts have generally demonstrated a predominance of c-ANCA/proteinase 3 (PR3) positivity, whereas the UAE cohort showed a higher prevalence of p-ANCA/myeloperoxidase (MPO)-positive disease, suggesting potential regional heterogeneity in serological patterns. The authors further proposed that these differences may reflect variations in immunogenetic and environmental factors, as well as differences in ANCA testing methodologies, underscoring the need for standardized testing and multicenter Gulf registries [13]. Given the small number of patients in our institutional registry, no conclusions regarding regional phenotypic or serological patterns can be drawn. Nevertheless, our cohort represents one of the few transplant-specific reports from Saudi Arabia, contributes valuable data from an underrepresented population, and complements the emerging epidemiological literature from the Gulf region while highlighting the need for larger multicenter collaborative studies.

Incidence and timing

Reported recurrence rates of AAV following kidney transplantation range from approximately 3% to 10%, with lower rates observed in contemporary cohorts, likely reflecting advances in immunosuppressive therapy [6,9,10]. Recurrence may occur at any time after transplantation, ranging from early relapse within weeks to late recurrence several years later. Early recurrence, although uncommon, is typically more aggressive and may reflect persistent subclinical disease activity at the time of transplantation [10-12]. In our case, recurrence occurred early and was associated with a severe clinical course.

In contrast, late recurrence typically follows a more indolent course, presenting with gradual deterioration in graft function, proteinuria, or active urinary sediment. These presentations may mimic other causes of graft dysfunction, including chronic rejection or calcineurin inhibitor toxicity, often necessitating histological confirmation [10,11].

Risk factors and diagnosis

Several factors have been proposed to influence post-transplant outcomes, including advanced recipient age, lower glomerular filtration rate, PR3-ANCA positivity at transplantation, and adverse histopathological features; however, their association with disease recurrence remains inconsistent across published studies [14]. Other factors, including shorter duration of remission prior to transplantation and ongoing subclinical disease activity, may also contribute to increased risk of recurrence [14-16].

Allograft biopsy remains essential for diagnosis, demonstrating pauci-immune necrotizing and/or crescentic glomerulonephritis in the appropriate clinical context [15]. In our case, an early biopsy was critical in establishing the diagnosis and guiding management.

Management and outcomes

Management strategies are extrapolated from native kidney disease and reported post-transplant recurrence cases and may include high-dose corticosteroids, rituximab, plasma exchange, and adjustment of maintenance immunosuppression according to disease severity [17,18]. Rituximab has demonstrated efficacy in recurrent disease and is increasingly used in selected patients [17,18]. However, intensification of immunosuppression increases the risk of infection, as observed in our patient. Despite initial response to therapy, outcomes may be poor, particularly in early and severe recurrence, and infectious complications may substantially contribute to morbidity and mortality.

Limitations

Several limitations should be acknowledged. First, this report combines a single case with a retrospective review of an institutional transplant registry, in which only six patients underwent kidney transplantation for AAV over an 18-year period. Consequently, the small sample size limits the generalizability of our findings and precludes meaningful statistical analysis. Second, the rarity of post-transplant AAV recurrence, together with the limited number of affected patients, restricts the ability to identify reliable predictors of recurrence or draw definitive conclusions regarding optimal management strategies. Third, follow-up duration varied among patients, and detailed longitudinal data, including serial ANCA titers and standardized disease activity assessments, were not consistently available for all cases. Finally, because this report is centered on a single severe recurrence, it may overrepresent the clinical impact of aggressive early post-transplant recurrence. Nevertheless, by combining a well-characterized case with institutional registry data, our study provides valuable real-world insight into the presentation, management, and outcomes of recurrent AAV following kidney transplantation in a regional transplant center.

## Conclusions

Kidney transplantation remains the preferred treatment for patients with ESKD secondary to AAV, with favorable graft outcomes observed in most patients when transplantation is performed during disease remission. Although recurrence is uncommon, it remains a significant clinical challenge and may occur despite apparent clinical remission, occasionally presenting with an aggressive course and poor outcomes, as illustrated by our case. This report highlights the importance of maintaining a high index of suspicion in patients with unexplained graft dysfunction, where early allograft biopsy is essential for timely diagnosis and management. Further multicenter studies are needed to better define risk factors and optimize preventive and therapeutic strategies in this population.
